# Lesions causing central sleep apnea localize to one common brain network

**DOI:** 10.3389/fnana.2022.819412

**Published:** 2022-09-29

**Authors:** Taoyang Yuan, Zhentao Zuo, Jianguo Xu

**Affiliations:** ^1^Department of Neurosurgery, West China Hospital, Sichuan University, Chengdu, China; ^2^State Key Laboratory of Brain and Cognitive Science, Institute of Biophysics, Chinese Academy of Sciences, Beijing, China; ^3^Hefei Comprehensive National Science Center, Institute of Artificial Intelligence, Hefei, China; ^4^University of Chinese Academy of Sciences, Institute of Biophysics, Chinese Academy of Sciences, Beijing, China

**Keywords:** central sleep apnea (CSA), lesion network mapping, focal brain lesions, neuroanatomical substrate, brain imaging

## Abstract

**Objectives:**

To characterize the specific brain regions for central sleep apnea (CSA) and identify its functional connectivity network.

**Methods:**

We performed a literature search and identified 27 brain injuries causing CSA. We used a recently validated methodology termed “lesion network mapping” to identify the functional brain network subtending the pathophysiology of CSA. Two separate statistical approaches, the two-sample *t*-test and the Liebermeister test, were used to evaluate the specificity of this network for CSA through a comparison of our results with those of two other neurological syndromes. An additional independent cohort of six CSA cases was used to assess reproducibility.

**Results:**

Our results showed that, despite lesions causing CSA being heterogeneous for brain localization, they share a common brain network defined by connectivity to the middle cingulate gyrus and bilateral cerebellar posterior lobes. This CSA-associated connectivity pattern was unique when compared with lesions causing the other two neurological syndromes. The CAS-specific regions were replicated by the additional independent cohort of six CSA cases. Finally, we found that all lesions causing CSA aligned well with the network defined by connectivity to the cingulate gyrus and bilateral cerebellar posterior lobes.

**Conclusion:**

Our results suggest that brain injuries responsible for CSA are part of a common brain network defined by connectivity to the middle cingulate gyrus and bilateral cerebellar posterior lobes, lending insight into the neuroanatomical substrate of CSA.

## Introduction

Central sleep apnea (CSA) is characterized by repeated episodes of airflow reduction or interruption due to a lack of drive to breathe during sleep (Javaheri and Dempsey, 2013; Orr et al., 2017; Dempsey, 2019). Because of this common disorder's management and effects, CSA has garnered a lot of attention recently (Baillieul et al., 2019). Clinically, CSA should be taken seriously because it can lead to a number of acute physiological effects, such as arterial oxygen desaturation, hypercapnia, postapneic awakenings, increase in ventilatory drive, and changes in arterial blood pressure (Hernandez and Patil, 2016; Baillieul et al., 2019). In addition, CSA has been associated with an increased risk of adverse cardiovascular outcomes and mortality (Eckert et al., 2007; Baillieul et al., 2019). Polysomnography recording is the main study for confirming the diagnosis of CSA (Javaheri and Dempsey, 2013). To date, early detection, diagnosis, and effective treatment of CSA also remain challenging.

The etiology of CSA includes heart failure, high altitudes, chronic opiate use, stroke, brainstem and spinal cord disorders, and chronic renal failure (Javaheri and Dempsey, 2013; Pevernagie et al., 2017). According to the pathophysiology, CSA syndromes can be subdivided into physiological central apnea, hypocapnia/eucapnia-related (nonhypercapnic) CSA, and hypercapnia-related CSA. Hypocapnia/eucapnia-related (nonhypercapnic) CSA may be caused by heart failure, high altitudes, chronic renal failure, or could be idiopathic when a specific trigger is not identified. Its main pathophysiological mechanism is the instability in ventilatory control, which can be explained by the two processes of high loop gain and narrow CO_2_ reserve (Hernandez and Patil, 2016; Orr et al., 2017). High loop gain results in greater ventilatory response and, consequently, hypocapnia. A narrow CO_2_ reserve is a condition in which the sleep eupneic CO_2_ set point is close to the sleep PaCO_2_ apnea threshold; as a result, a smaller change in sleep PaCO_2_ may cross the apnea threshold and lead to CSA (Khan et al., 2005). Hypercapnia-related CSA occurs in disorders in which there is alveolar hypoventilation because of an absent or diminished wakefulness stimulus to breathe, such as opioid toxicity, neuromuscular disorders, and brainstem and spinal cord lesions/disorders. As mentioned above, various pathological processes in the brainstem, such as compression, edema, ischemia, infarction, tumors, encephalitis, and neurodegenerative disorders, may lead to CSA, because central chemoreceptors and respiratory centers are located in this region (Javaheri and Dempsey, 2013; Pevernagie et al., 2017). For example, the pre-Bötzinger complex (preBötC), an important neural network responsible for the regulation of respiratory rhythm, is located in the brain stem, particularly in the ventrolateral medulla. The functional activation of this area increases the inspiratory frequency, while inhibition leads to apnea (Cregg et al., 2017). Opioids lead to CSA mainly by inhibiting the pre-Bötzinger complex (Pevernagie et al., 2017). While the brainstem centers that regulate breathing (particularly the pons-medulla) are well-understood, the role of the cortex in modulating breathing during sleep is poorly understood (Nachtmann et al., 1995; Siccoli et al., 2008; Kim et al., 2018; Huhtakangas et al., 2020). We hypothesize that, when CSA is determined by brain injuries outside the brainstem, an impairment of a broader network that involves both cortical and subcortical regions can be expected.

In this study, we adopted a novel methodology called “lesion network mapping” to identify a possible common functional network on the basis of CSA. This technology has been used for multiple neurological disorders, such as auditory hallucinations, aphasia, parkinsonism, freezing of gait, and free will (Fox, 2018).

## Materials and methods

### Case selection

The lesions causing CSA were identified through a search of studies published in English in the PubMed database using the terms [(sleep apnoea OR sleep apnea) AND (lesion OR tumor OR tumor OR stroke OR infarct OR hemorrhage OR hemorrhage OR bleeding OR traumatic OR damage OR injury) AND (brain OR cerebral) AND (case report OR case series)] on 29 March 2021, and 180 studies were found. The inclusion criteria were as follows: (i) CSA diagnosis, (ii) high-resolution CT or MR images, and (iii) CSA due to brain lesions. The exclusion criteria were as follows: (i) epidural brain tissue compression and (ii) previous CSA history. Finally, 24 studies consisting of 27 patients met our criteria and were included.

### Lesions

Brain injuries responsible for CSA were traced onto a standardized human brain atlas [2 × 2 × 2 mm^3^ Montreal Neurological Institute (MNI) space] by hand using MRIcron software (https://www.mccauslandcenter.sc.edu/crnl/tools). The lesion in each slice of the CT or MRI was traced, and neuroanatomical landmarks were used to accurately transfer the lesion location onto the brain template.

### Lesion network mapping

Relative to voxel-based lesion-symptom mapping, lesion network mapping can extend traditional methods by mapping lesions to circuits using an external dataset of resting-state functional MRI (rs-fMRI) scans derived from a large sample of healthy subjects, but not rs-fMRI data obtained from the patient with the lesion (Joutsa et al., 2022). Because the brain tissue at the lesion site is lost, it is no longer functionally connected to any brain region. Compared with lesion network maps from lesions not causing this symptom, network maps specific to the symptom can be identified.

In this study, rs-fMRI data for 1,083 healthy subjects were downloaded from the open-access Human Connectome Project (HCP) S1200 data release (Glasser et al., 2013; Van Essen et al., 2013). For the rs-fMRI data preprocessing, we utilized detrending, bandpass filtering, and global signal regression to determine connectivity. The network of brain regions functionally connected to each lesion location was calculated according to previous studies (Boes et al., 2015; Joutsa et al., 2018). First, traced lesions were used as a seed region. Then, rs-fMRI data from the healthy subjects were used to determine the connectivity between each lesion location and all other brain voxels, including both positive and negative correlations (anticorrelations) with the lesion region. Second, a binarized whole lesion network map was created for each lesion using a threshold at T = ±5 (uncorrected, *p* < 6 × 10^−7^). Finally, the binary lesion network map for each lesion was overlaid, and the number of cases with overlapping networks in each voxel was calculated to determine common network sites across the lesions.

### Specificity and conjunction analyses, and the definition of the CSA lesion network

To assess the specificity of our findings, we compared them to two “control” datasets of non-CSA-causing lesions: lesions related to criminal behavior (*n* = 17) and coma (*n* = 12) from previous studies (Fischer et al., 2016; Darby et al., 2018). To ensure that the results were robust to different analysis methods, we used two separate statistical approaches, including a two-sample *t*-test using continuous connectivity scores and a Liebermeister test using binarized scores. Two-sample *t*-tests were implemented in Statistical Parametric Mapping software (SPM12; http://www.fil.ion.ucl.ac.uk/spm/software/spm12/), and a strict threshold (FWE-corrected *p* < 0.05) was considered statistically significant. The Liebermeister test was carried out using nonparametric mapping (http://www.mccauslandcenter.sc.edu/mricro/npm/), with the correlated (*T* ≥ 5) and anticorrelated (*T* ≤ −5) networks being thresholded and analyzed separately, as previously reported (Kim et al., 2021). The analyses were performed on the whole brain, disregarding voxels affected in < 10% of all cases (Karnath et al., 2018). Two thousand permutations and FWE correction for multiple comparisons (FWE-corrected *p* < 0.05) were performed. To determine the specific regions for CSA, we overlapped the findings of our four specificity tests, and the voxels surviving in all four tests were acquired. Then, we performed a conjunction analysis between voxels surviving in all four specificity tests and lesion network mapping for CSA. Only three positively correlated regions of interest (ROI) survived, in the cingulate gyrus and bilateral cerebellar posterior lobes. We define a distributed brain network with strong connectivity with these regions that encompass our lesion locations, causing CSA. In acquiring the network, we calculated the positive connectivity between the voxels in other brain sites and the regions, including the cingulate gyrus and bilateral cerebellar posterior lobes.

Furthermore, to verify the reliability of the merging network, we examined the relationship between all lesions and the network mapping of CSA.

### Reproducibility

Ondine's curse (OC), also known as central hypoventilation syndrome or CSA, is a phenomenon characterized by episodes of repeated apnea during sleep due to disorders of the central nervous system (Zaidi et al., 2018). A second replication cohort of CSA patients was generated through a second literature search of studies in the PubMed database using the terms [(Ondine's curse OR central hypoventilation syndrome) AND (lesion OR tumor OR tumor OR stroke OR infarct OR hemorrhage OR hemorrhage OR bleeding OR traumatic OR damage OR injury) AND (brain OR cerebral) AND (case report OR case series)]. Records included in the previous search were excluded. Finally, an additional six patients from five studies were enrolled, and lesion network mapping was repeated. Subsequently, we overlaid these two lesion network mappings. To identify the specificity of this network, we compared this network pattern with the network from lesions causing coma using two-sample *t*-tests. A voxel-based FWE-corrected *p* < 0.05 was considered statistically significant.

## Results

### Lesion analysis

**Figure 1:**
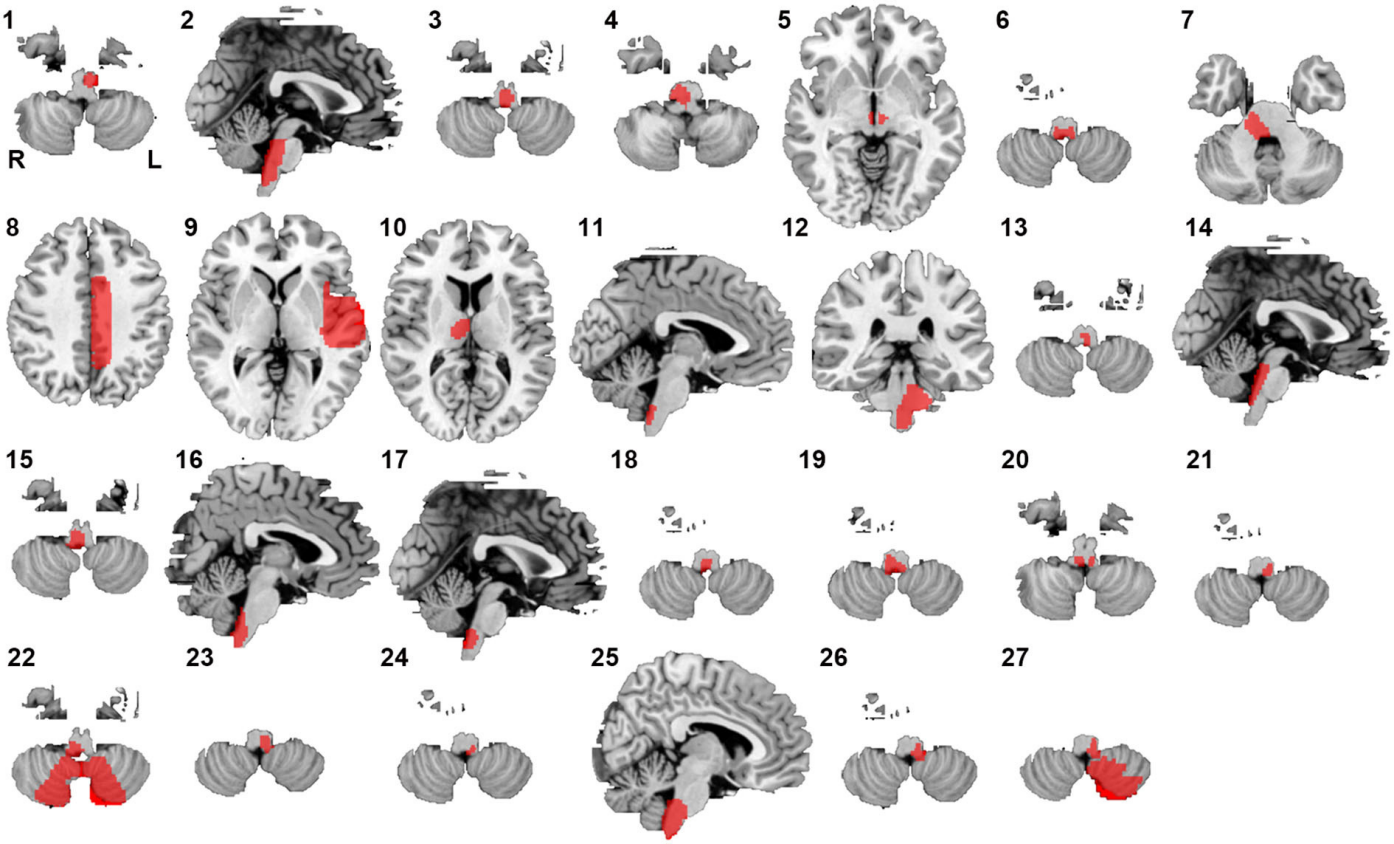
Lesions causing CSA. Twenty-seven lesions causing CSA were mapped onto a standardized brain atlas.

**Figure 2:**
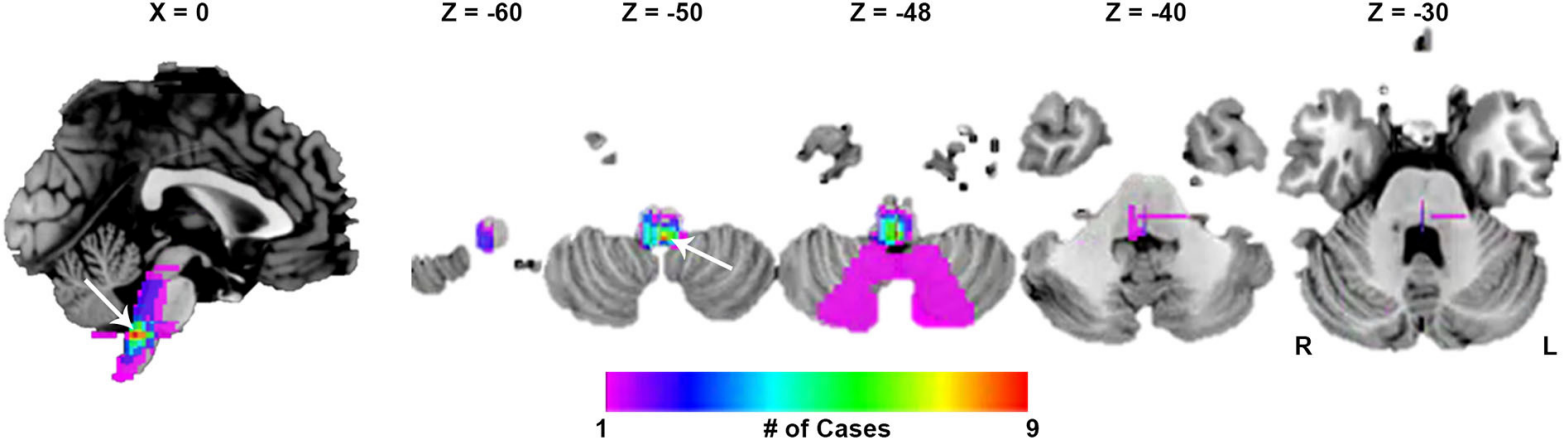
Lesion overlap maps. The peak overlap was 9/27 (33.3%) and occurred in the dorsal medulla (white arrow).

Twenty-seven lesions causing CSA were identified in the present study. We found 24 lesions located in the brainstem, 1 lesion in the basal ganglia, and 2 lesions in the cerebral cortex (Figure 1). CSA-causing lesions maximally overlapped in the dorsal medulla (9 of 27 cases; Figure 2). Detailed information about the patients with lesions causing CSA is summarized in Supplementary Table 1.

### Deriving a CSA network from reported brain lesions

**Figure 3:**
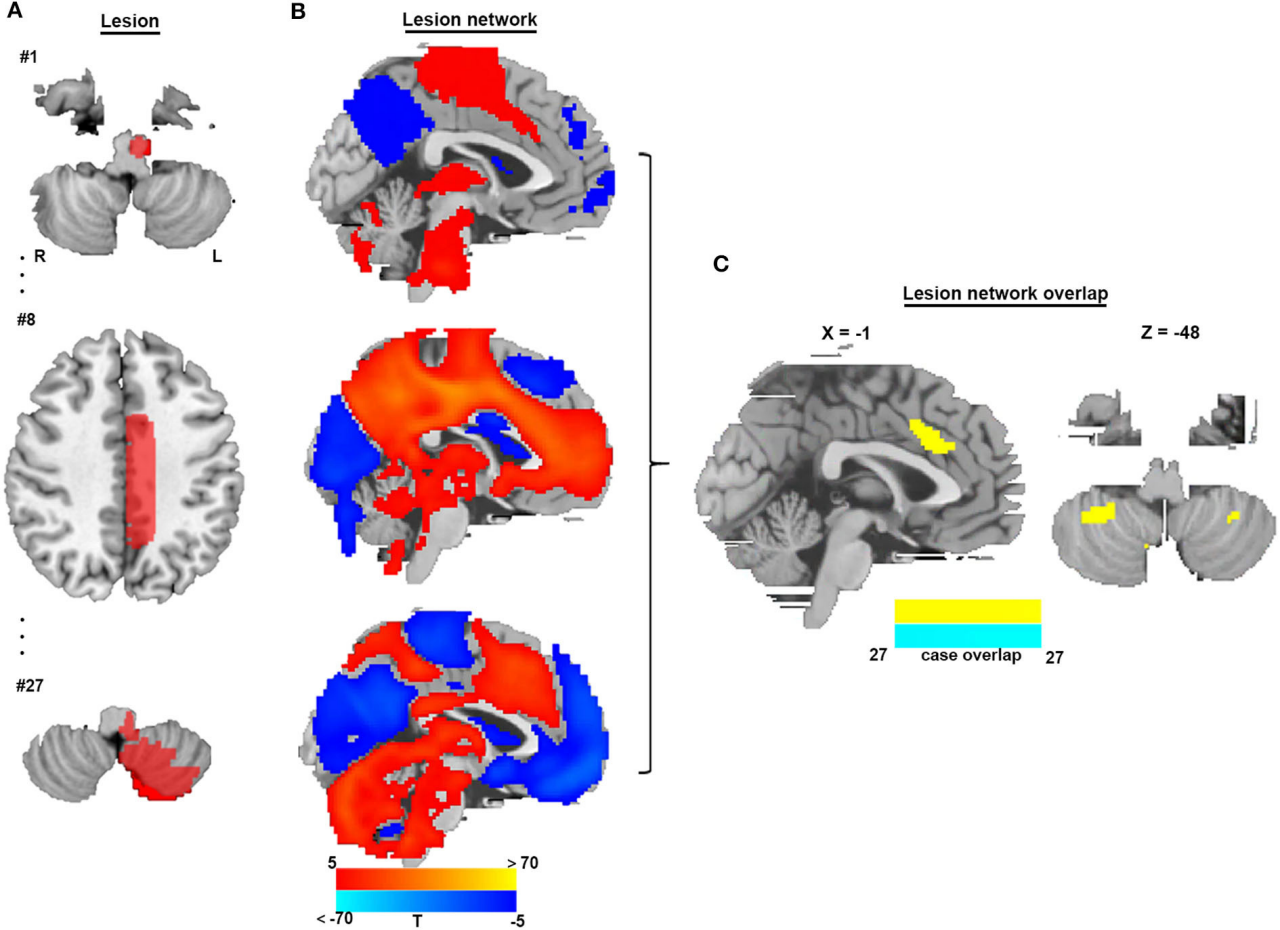
Lesion network mapping of CSA. **(A)** Three representative lesions of a total of 27 were traced onto a standardized MNI brain template. **(B)** Brain regions functionally connected to each lesion location were calculated using a large resting-state functional connectivity database from HCP (*n* = 1,083). **(C)** Overlap of thresholded functional connectivity maps (*t* ≥ |5|) from each lesion-identified brain region connected to all lesion locations.

**Figure 4:**
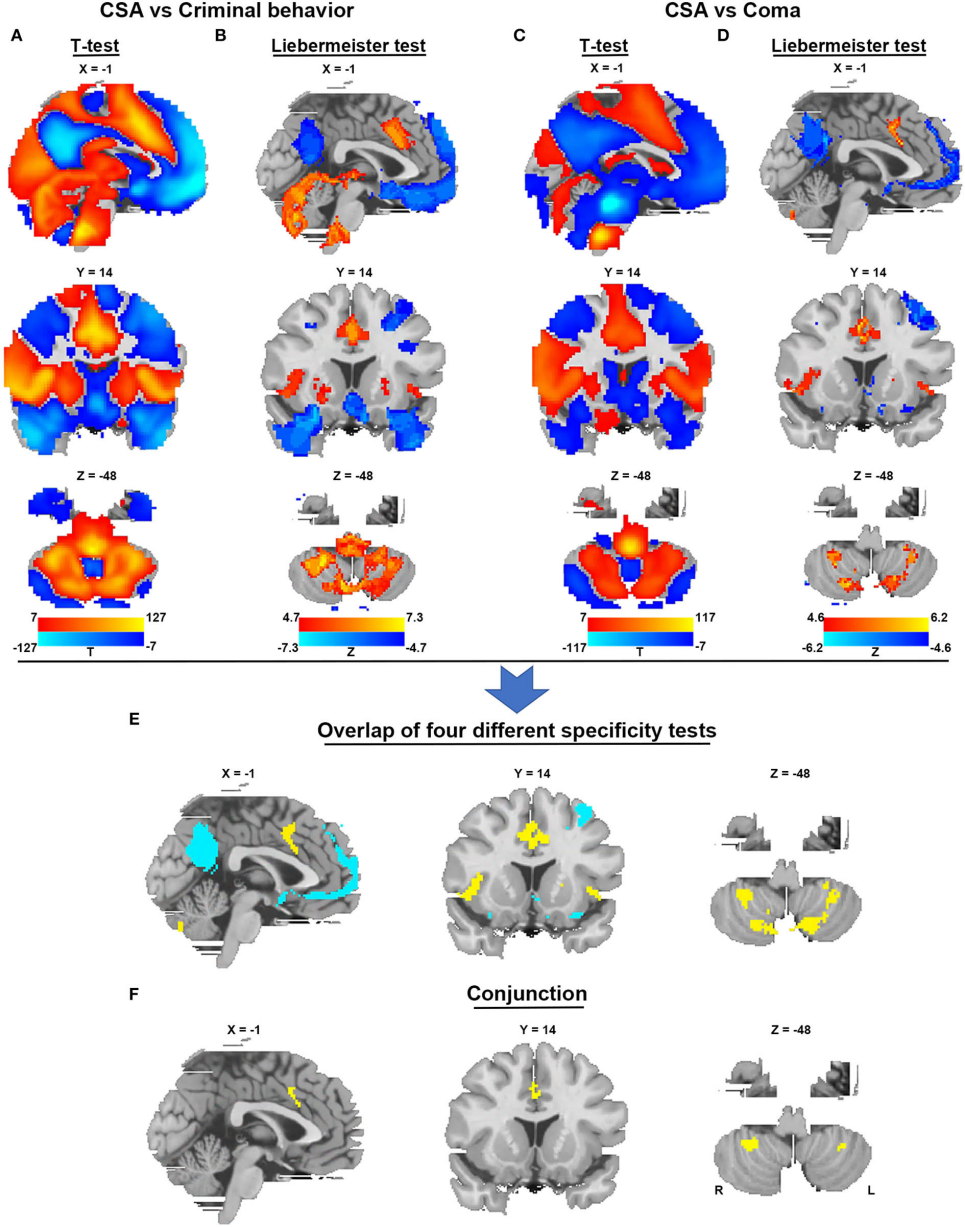
Specificity and conjunction analysis. **(A,B)** Comparison of the network of CSA with the network of lesions causing criminal behavior using two-sample *t*-tests and the Liebermeister test. **(C,D)** Comparison of the network of CSA with the network of lesions causing coma using two-sample *t*-tests and the Liebermeister test. **(E)** Overlap of four different specificity tests to identify connections specific to lesions causing CSA relative to control lesions. **(F)** A conjunction analysis showed that regions in the middle cingulate gyrus and bilateral cerebellar posterior lobes were specific to lesions causing CSA.

To identify brain regions in the CSA network, we performed functional connectivity analysis using rs-fMRI data from 1,083 healthy subjects. Figure 3 shows that all CSA-causing lesions were positively connected to the cingulate gyrus, bilateral cerebellum, and bilateral thalamus and negatively connected to the bilateral middle occipital gyrus. To determine the specific regions responsible for CSA, we compared this connectivity pattern specific to CSA-causing lesions with connectivity patterns for two other neurological syndromes, criminal behavior and coma, using two separate statistical approaches (Figures 4A–D). Figure 4E demonstrates the specificity of the network from CSA-causing lesions relative to the two other neurological syndromes. The conjunction analysis found that the cingulate gyrus and bilateral cerebellar posterior lobes are the regions specific to CSA (Figure 4F; Supplementary Table 2).

### Replication from an additional independent cohort of six CSA cases

**Figure 5:**
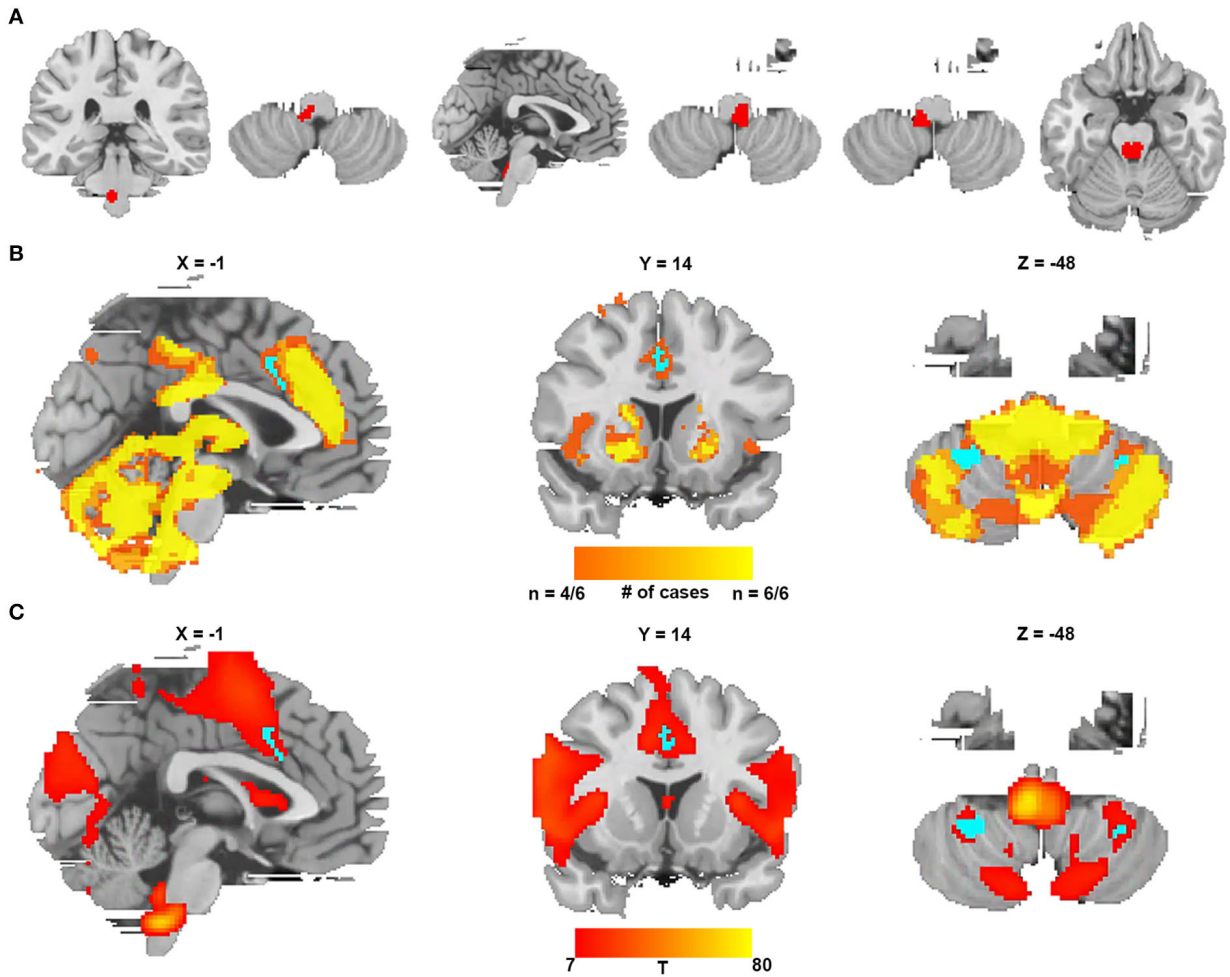
Lesion network mapping from a replication cohort of six lesions causing CSA. **(A)** Six lesions causing CSA were traced onto a standardized MNI brain template. **(B)** Lesion network overlap map from each lesion showing regions functionally connected to the greatest number of lesion locations (the regions specific to CSA are blue). **(C)** The *t*-test comparing the functional connectivity of the replication cohort with lesions causing coma (voxelwise FWE-corrected *P* < 0.05) (the regions specific to CSA are blue).

We identified six additional cases causing CSA after expanding the search criteria. The lesions from these six cases were located in different parts of the brainstem, as shown in Figure 5A and Supplementary Table 3. Figure 5B shows the lesion network mapping derived from the replication cohort, with 4/6 lesions associated networks overlapping in the cingulate gyrus and bilateral cerebellar posterior lobes. Figure 5C demonstrates a difference map by comparing with the networks from lesions causing coma. The middle cingulate gyrus and bilateral cerebellar posterior lobes also survived.

### Connectivity with the identified regions in the middle cingulate gyrus and bilateral cerebellar posterior lobes is specific to lesions causing CSA

**Figure 6:**
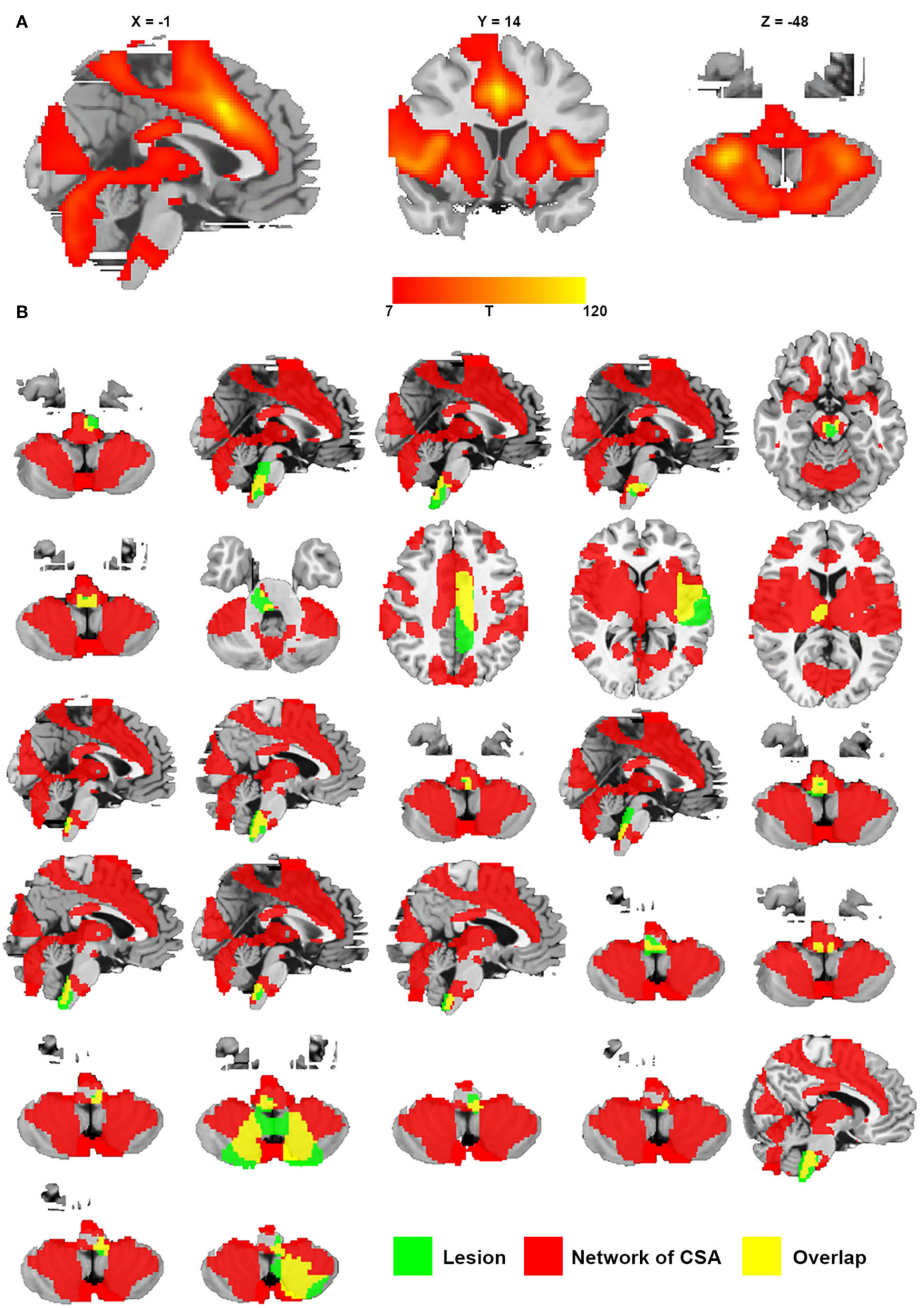
Network of CSA defined by connectivity to the middle cingulate gyrus and bilateral cerebellar posterior lobes. **(A)** Resting-state functional connectivity with the site of the middle cingulate gyrus and bilateral cerebellar posterior lobes. **(B)** All lesions causing CSA were located in this network (green represents lesions causing CSA; red represents the network of CSA; and yellow represents the overlap of the lesion and the network).

We defined a network that encompasses lesion locations causing CSA through connectivity with the positive regions of the middle cingulate gyrus and bilateral cerebellar posterior lobes. Figure 6A shows the network mapping for CSA by calculating the functional connectivity between the specific regions for CSA and all other brain voxels. Notably, we found that all lesions causing CSA fell within this network mapping, as displayed in Figure 6B.

## Discussion

There are three significant findings in this study. First, our results suggest that although CSA-causing lesions occur in different brain locations, they share a common brain network defined by connectivity to the middle cingulate gyrus and bilateral cerebellar posterior lobes. Second, this network is specific to lesions causing CSA compared to control lesions. Third, these results are reproducible, which we validated in an independent second cohort of patients with CSA.

In recent years, an increasing number of researchers have noted that lesions in brain regions other than the brainstem, including cerebral infarction, tumor, and malformation, can also lead to CSA (Hermann and Bassetti, 2009; Lee et al., 2013; Zaffanello et al., 2017). Little is known about the potential neural mechanism of CSA due to a lack of effective and reasonable research methods. Expanding the awareness of CSA and understanding its underlying neuroanatomical substrate will facilitate the early diagnosis of CSA, improve the comprehensive management of patients, and develop new treatment options. Lesion network mapping, first proposed by the Michael D. Fox team, can be applied to identify the specific functional network for neurological syndromes based on the rs-fMRI data of healthy subjects; this method sets the location of the lesion reported to cause the neurological syndrome as the seed and has been successfully applied to a variety of neurological syndromes (Fox, 2018). Expanding awareness of CSA and understanding its underlying neuroanatomical substrates will facilitate an early diagnosis of CSA, improve the comprehensive management of patients, and suggest new treatment options. Through lesion network mapping, we found that all CSA-causing lesions, regardless of location, exhibited positive connectivity with the cingulate gyrus, bilateral cerebellum, and bilateral thalamus and negative connectivity with the bilateral middle occipital gyrus. To verify that these regions are specific for causing CSA, we selected all coma-causing lesions located in the brainstem and almost all crime-causing lesions located in the cerebral cortex as controls. The conjunction analysis revealed that only the CSA lesion network had strong connectivity with the middle cingulate gyrus and bilateral cerebellar posterior lobes.

In sleep research, the role and functional changes of the cerebellum are of increasing interest. With the help of electroencephalography (EEG), fMRI, positron emission tomography (PET), and the combination of these technologies, researchers have found that cerebellar activity and cortico-cerebellar connectivity are sleep-stage-dependent, and the cerebellum also plays a regulatory role in the sleep process (Canto et al., 2017). In contrast to decreased activity during nonrapid eye movement stages (NREM), cerebellar activity increases during rapid eye movement (REM). Moreover, previous data indicate that sleep disorders and cerebellar pathology are intricately involved with each other (DelRosso and Hoque, 2014; Canto et al., 2017). For example, patients with spinocerebellar ataxias, which are characterized by degeneration of the cerebellum and its afferent and efferent connections, show a variety of sleep disorders, such as restless leg syndrome (RLS), REM behavior disorder (RBD), excessive daytime sleepiness (EDS), and sleep apnea (Kumar et al., 2008; Silva et al., 2016; Martinez et al., 2017). In the present study, we found that the cerebellum is a key network node of CSA. Combined with our results and previous research findings (cerebellar activity is sleep-stage-dependent), the possible reason for the occurrence of apnea in the sleep stage is the dependence of cerebellar activity on the sleep stage. Unfortunately, due to the lack of our own CSA patients, we cannot further study whether there is a direct relationship between the time of CSA occurrence and sleep stages. More importantly, previous studies have also suggested that the cingulate gyrus and cerebellum play significant roles in central respiratory control (Lee et al., 2013; Macey et al., 2016; Sacchetti et al., 2017). In respiration, the cerebellum has the ability to respond to hypercapnia and coordinate the respiratory musculature, and the cingulate gyrus can mediate the emotional drive for inspiration from shortness of breath (Patwari et al., 2010; Lee et al., 2013; Sacchetti et al., 2017). Case reports have shown that CSA can be secondary to pure cerebellar dysplasia or atrophy observed from MRI data (Qingyu et al., 2005; Taytard et al., 2020). In congenital central hypoventilation syndrome, structural and functional MRI and limited postmortem studies reveal abnormalities in both the cingulate gyrus and the cerebellum (Kinney, 2008; Kumar et al., 2008, 2010; Patwari et al., 2010; Sharman et al., 2014). Clinically, researchers found that CSA is a common symptom in patients with Chiari I malformation with inferior cerebellar hernia into the foramen magnum rather than the brainstem (Zaffanello et al., 2017). Even surgery involving the cerebellum without brainstem invasion can also lead to CSA (Lee et al., 2013). These results suggest that the cerebellum and cingulate gyrus may play a direct regulatory role in CSA. This study also sheds light on the role of the cingulate gyrus and cerebellum in central respiratory control. It is possible that abnormal functional connectivity between the cingulate gyrus and bilateral cerebellar posterior lobes is the neuroanatomical substrate leading to CSA. Thus, we defined the connectivity between the positive regions of the middle cingulate gyrus and the bilateral cerebellar posterior lobes as the core functional network for CSA in the present study.

In the network mapping of CSA, we found that the medulla was one of the important nodes. A previous study using retrograde tracing on rats to investigate connections with the rostral ventral respiratory cell group reported a close nerve fiber connection between the cerebellar/cingulate gyrus and this cell group (Gaytán and Pásaro, 1998). Located in the ventrolateral part of the medulla oblongata in rats, the rostral ventral respiratory cell group contains the structures necessary for respiratory rhythm generation (Hayakawa et al., 2004). Our results suggested that the middle cingulate gyrus, cerebellum posterior lobes, and medulla oblongata co-regulate CSA. The cingulate gyrus and posterior cerebellum lobes on one side and the medulla oblongata on the other side might represent the cortical and subcortical centers of CSA, respectively. In our study, overlapping lesions showed that nine of the 27 lesions were located in the dorsal medulla, while the majority of the lesions were located in the brainstem. Only one or two patients had lesions involving the cingulate gyrus and the posterior lobes of the cerebellum. A possible reason for the higher probability of CSA in brainstem injury than in the cingulate gyrus and cerebellum is that brainstem plasticity is worse than that observed in other brain regions of the CSA network. In addition, we also found that the bilateral thalamus and insula were included in the CSA network. The insular cortex of mammals plays a crucial role in autonomic regulation, such as heart rate and respiratory rate (Sanchez-Larsen et al., 2021). Several studies have demonstrated that respiratory responses can be elicited by stimulation of the insular cortex in different mammalian species (Aleksandrov et al., 2000; Bagaev and Aleksandrov, 2006), and seizures or lesions in the insula also have a certain probability of causing CSA (Hermann et al., 2007). The thalamus is another supratentorial brain structure that modulates the autonomic nervous system (Brunyé et al., 2021). Multiple studies have reported that acute stroke in the thalamus can induce sleep-related breathing alterations, including CSA and obstructive sleep apnea syndrome (Hermann et al., 2007). Gaytán and Pásaro (1998) also found that there are structural fiber connections between the thalamus (thalamic posterior nuclear group, subparafascicular, parafascicular, gelatinosus thalamic nuclei, and subthalamic nuclei)/insula (granular and agranular insular cortex) and the rostral ventral respiratory cell group. In the 27 lesions causing CSA, we found parts of these lesions were located in the thalamus and the insula. Therefore, we have enough reasons to believe that the thalamus and insula may be involved in the CSA network. We investigated whether the CSA-causing lesions were located in this network by overlapping the network map of CSA with individual lesions and found that all CSA-causing lesions were well-aligned with the network defined by connectivity to the cingulate gyrus and bilateral cerebellar posterior lobes. This finding further confirms the specificity of the CSA network, defined by positive connectivity to the middle cingulate gyrus and bilateral cerebellar posterior lobes.

To further establish the reliability of our results, we repeated the same analysis with the second cohort of CSA-causing lesions and found that the previously positive areas in the middle cingulate gyrus and bilateral cerebellum posterior lobes were in good agreement with the results of the replication cohort. To validate the specificity of the CAS-causing network from the replication cohort, 12 coma-causing brainstem lesions previously published by the Michael D. Fox team (Fischer et al., 2016) were chosen as the control to eliminate the potential impact of lesion location centralization on the analysis results. The specific regions for the network of CSA, the middle cingulate gyrus and bilateral cerebellum posterior lobes, also survived in the functional connectivity of the replication cohort vs. lesions causing coma. This replication increases our confidence that CSA-causing lesions share a common brain network defined by connectivity to the cingulate gyrus and bilateral cerebellar posterior lobes.

The current study has several limitations. First, all patients with lesions causing CSA from the literature in PubMed were prone to publication bias, and there were no cases from our own database to further confirm this finding. The accuracy of lesion tracing is limited by the quality of the published images, and the lesion locations were determined according to the neuroanatomical landmarks. Second, our systematic search may not include all the lesions causing CSA due to different definitions of keywords by authors. Third, the distortion of normal anatomic structures caused by tumors in the brainstem may lead to deviations in tracking lesions. Fourth, due to a lack of functional neuroimaging data from patients themselves, we are unable to investigate the direct functional abnormality of the brain caused by lesions. Fifth, in the present study, due to the lack of our own patients, it is difficult to verify the role of this specific network in different CSA physiological periods.

## Conclusion

The lesions causing CSA share a common brain network defined by connectivity to the middle cingulate gyrus and bilateral cerebellar posterior lobes. The middle cingulate gyrus and bilateral posterior cerebellar lobes are potentially key regions for CSA, which has potential implications for our understanding of the neuroanatomical substrate of CSA. Our findings may provide a new and testable therapeutic target for CSA. In follow-up studies, such as animal experiments, the middle cingulate gyrus and bilateral cerebellar posterior lobes can be used as potential research targets for CSA therapy.

## Data availability statement

The original contributions presented in the study are included in the article/Supplementary material, further inquiries can be directed to the corresponding author/s.

## Ethics statement

We did not include original data from any studies with human participants or animal studies performed by any of the authors. Lesions causing CSA were identified from the literature in the PubMed database. Rs-fMRI data were provided in part by the Human Connectome Project, WU-Minn Consortium (Principal Investigators: David Van Essen and Kamil Ugurbil; 1U54MH091657).

## Author contributions

TY, ZZ, and JX: design and conceptualized study and interpreted the data. TY: major role in the acquisition of data. TY and ZZ: analyzed the data and drafted the manuscript for intellectual content. ZZ and JX: revised the manuscript for intellectual content. All authors contributed to the article and approved the submitted version.

## Funding

Data were provided in part by the Human Connectome Project, WU-Minn Consortium (Principal Investigators: David Van Essen and Kamil Ugurbil; 1U54MH091657) funded by the 16 NIH Institutes and Centers that support the NIH Blueprint for Neuroscience Research; and by the McDonnell Center for Systems Neuroscience at Washington University. This work was financially supported by the 1.3.5 Project for Disciplines of Excellence, West China Hospital, Sichuan University (ZYJC18007), the Ministry of Science and Technology of China grant (2020AAA0105601), the University Synergy Innovation Program of Anhui Province, China (GXXT-2021-002), and the Youth Innovation Promotion Association CAS (2021091).

## Conflict of interest

The authors declare that the research was conducted in the absence of any commercial or financial relationships that could be construed as a potential conflict of interest.

## Publisher's note

All claims expressed in this article are solely those of the authors and do not necessarily represent those of their affiliated organizations, or those of the publisher, the editors and the reviewers. Any product that may be evaluated in this article, or claim that may be made by its manufacturer, is not guaranteed or endorsed by the publisher.
