# Supplementary material for: Lesions causing central sleep apnea localize to one common brain network
Source: Front Neuroanat. 2022 Sep 29;16:819412. doi: 10.3389/fnana.2022.819412 (PMC9559371; doi:10.3389/fnana.2022.819412)
Supplement: Supplementary file 1 [file Data_Sheet_1.docx]

**Lesions causing central sleep apnea localize to one common brain network**

**Taoyang Yuan^1^, Zhentao Zuo^2, 3, 4*^, Jianguo Xu^1*^**

^1^ Department of Neurosurgery, West China Hospital, Sichuan University, Chengdu, China.

^2^ State Key Laboratory of Brain and Cognitive Science, Institute of Biophysics, Chinese Academy of Sciences, Beijing, China

^3^ Hefei Comprehensive National Science Center, Institute of Artificial Intelligence, Hefei, China

^4^ University of Chinese Academy of Sciences, Institute of Biophysics, Chinese Academy of Sciences, Beijing, China

**Supplementary Table 1**. Central sleep apnea cases from literatures.

| Case | Citation | Age/sex | Lesion type | Lesion location | Images |
| --- | --- | --- | --- | --- | --- |
| 1 | Sakurai N,1993[1] | 50/M | Unknown | Inferior olivary nuclei | MR |
| 2 | Agitani-Shimono Kuriko, 2011[2] | 17/F | Cavernous hemangioma | Dorsal pontine and medulla oblongata | MR |
| 3 | Fujimoto K,2018[3] | 12/F | Tumor | Medulla oblongata | MR |
| 4 | Haley MD,2019[4] | 70/M | Aneurysm | Pontomedullary  junction | MR |
| 5 | Faludi B,2016[5] | 62/F | Infarction | Bilaterally mesencephalon and paramedian thalamus | MR |
| 6 | Yasaki E,2001[6] | 3/F | Leigh syndrome | Medulla oblongata | MR |
| 7 | Heckmann JG,2014[7] | 62/M | Infarction | Right cerebellar peduncle | MR |
| 8 | Hermann DM,2007[8] | 49/M | Infarction | Left cingulate cortex | MR |
| 9 | Hermann DM,2007[8] | 52/M | Infarction | Left insula and gyrus angularis | MR |
| 10 | Hermann DM,2007[8] | 68/M | Infarction | Right  paramedian thalamus | MR |
| 11 | Kyoshima K,2004[9] | 55/M | Surgery | Left medulla oblongata | MR |
| 12 | Kuhn M,1999[10] | 32/M | Tumor | Brainstem | MR |
| 13 | Fujisawa H,2005[11] | 2/F | Surgery | Cervicomedullary  region | MR |
| 14 | Tatsumi C,1988[12] | 46/M | Mitochondrial encephalomy-opathy | Bilateral globi pallidi, from midbrain to medulla | MR |
| 15 | Manning HL,2000[13] | 18/F | Tumor | Medulla | MR |
| 16 | Wong CW,1992[14] | 34/M | Tumor | Medulla | MR |
| 17 | Funakawa I,1993[15] | 52/M | Multiple sclerosis | Medulla oblongata | MR |
| 18 | Funakawa I,1993[15] | 48/F | Multiple sclerosis | Tegmentum of  the medulla oblongata | MR |
| 19 | Marin-Sanabria EA,2006[16] | 52/F | Tumor | Medulla oblongata | MR |
| 20 | Ramar K.2009[17] | 66/F | Unknown | Bilateral medulla | MR |
| 21 | Pedroso JL,2009[18] | 79/F | Infarction | Left medulla | MR |
| 22 | Ho HT,2005[19] | 62/M | Infarction | Right medulla oblongata and  bilateral cerebellum | MR |
| 23 | Kapnadak SG,2010[20] | 72/F | Capillary  telangiectasia | Left medulla | MR |
| 24 | Nogues M S,2000[21] | 40/- | Syringobulbia | Left medulla | MR |
| 25 | Habek M,2009[22] | 37/M | Leptospirosis | Medulla oblongata  and caudal pons | MR |
| 26 | Mendoza M,2013[23] | 60/M | Infarction | Left medulla | MR |
| 27 | Fiedler E,2020[24] | 68/M | Infarction | Left medulla | MR |

M = male; F = female; - represents no report.

**Supplementary Table 2.** The coordinates (MNI space) of center of gravity within the conjunction of lesion network map.

| Voxels | x | y | z | Regions |
| --- | --- | --- | --- | --- |
| 48 | 1 | 15 | 37 | Bilateral middle cingulate gyrus |
| 74 | 29 | -48 | -50 | Right cerebellar posterior lobe |
| 14 | -34 | -50 | -48 | Left cerebellar posterior lobe |

**Supplementary Table 3**. Replication cohort of central sleep apnea from literatures.

| Case | Citation | Age/sex | Lesion type | Lesion location | Images |
| --- | --- | --- | --- | --- | --- |
| 1 | Schestatsky P,2004[25] | 55/M | Stroke | medulla | MR |
| 2 | Bogousslavsky J, 1990[26] | 65/M | Infarction | medulla | MR |
| 3 | Armangue T,2012[27] | 4/F | Dysgenesis | Brainstem | MR |
| 4 | Mishina M,2014[28] | 78/M | Infarction | medulla | MR |
| 5 | Mishina M,2014[28] | 72/M | Infarction | medulla | MR |
| 6 | Dooling EC,1977[29] | 5/M | Unknown | midbrain | Horizontal sections of the brain |

M = male; F = female; - represents no report.

**Reference**

[1] N. Sakurai, Y. Koike, Y. Kaneoke, T. Yasuda, and A. Takahashi, Sleep apnea and palatal myoclonus in a patient with neuro-Behcet syndrome. Internal medicine (Tokyo, Japan) 32 (1993) 336-9.

[2] K. Kagitani-Shimono, K. Kato-Nishimura, T. Okinaga, I. Mohri, N. Tachibana, K. Ozono, and M. Taniike, Long-term observation of absence of REM sleep caused by pontine cavernous hemangioma. Sleep Med 12 (2011) 1045-6.

[3] K. Fujimoto, H. Kasai, R. Kunii, J. Terada, and K. Tatsumi, Obstructive Sleep Apnea in a Severely Obese Child With Combined Central Sleep Apnea and Sleep-Related Hypoventilation Disorder Caused by a Medullary Tumor. J Clin Sleep Med 14 (2018) 1071-1074.

[4] M.D. Haley, D.B.H. Henderson, M. Nowell, W.M. Adams, and P.C. Whitfield, Giant vertebrobasilar aneurysm: a rare cause of central sleep apnoea. Br J Neurosurg 33 (2019) 559-561.

[5] B. Faludi, M. Toth, G. Pusch, and S. Komoly, Dynamic changes in sleep-related breathing abnormalities in bilateral paramedian mesencephalon and thalamus stroke: a follow-up case study. Sleep Breath 20 (2016) 237-42.

[6] E. Yasaki, Y. Saito, K. Nakano, H. Katsumori, K. Hayashi, T. Nishikawa, and M. Osawa, Characteristics of breathing abnormality in Leigh and its overlap syndromes. Neuropediatrics 32 (2001) 299-306.

[7] J.G. Heckmann, and S. Ernst, Central alveolar hypoventilation (Ondine's curse) caused by megadolichobasilar artery. Journal of stroke and cerebrovascular diseases : the official journal of National Stroke Association 23 (2014) 390-2.

[8] D.M. Hermann, M. Siccoli, P. Kirov, M. Gugger, and C.L. Bassetti, Central periodic breathing during sleep in acute ischemic stroke. Stroke 38 (2007) 1082-4.

[9] K. Kyoshima, K. Sakai, T. Goto, A. Tanabe, A. Sato, H. Nagashima, and J. Nakayama, Gross total surgical removal of malignant glioma from the medulla oblongata: report of two adult cases with reference to surgical anatomy. Journal of clinical neuroscience : official journal of the Neurosurgical Society of Australasia 11 (2004) 75-80.

[10] M. Kuhn, M. Lutolf, and W.H. Reinhart, The eye catcher. Ondine's curse. Respiration 66 (1999) 265.

[11] H. Fujisawa, Y. Yoshida, Y. Niida, M. Hasegawa, and J. Yamashita, Cyanotic breath-holding spell: a life-threatening complication after radical resection of a cervicomedullary ganglioglioma. Pediatric neurosurgery 41 (2005) 93-7.

[12] C. Tatsumi, M. Takahashi, S. Yorifuji, Y. Nishikawa, M. Kitaguchi, S. Hashimoto, and S. Tarui, Mitochondrial encephalomyopathy with sleep apnea. European neurology 28 (1988) 64-9.

[13] H.L. Manning, and J.C. Leiter, Respiratory control and respiratory sensation in a patient with a ganglioglioma within the dorsocaudal brain stem. Am J Respir Crit Care Med 161 (2000) 2100-6.

[14] C.W. Wong, Y.Y. Wai, T.N. Lui, and C.N. Chang, Bilateral glossopharyngeal neuralgia after excision of a solitary cervico-medullary haemangioblastoma: case report. Acta neurochirurgica 114 (1992) 64-7.

[15] I. Funakawa, K. Hara, T. Yasuda, and A. Terao, Intractable hiccups and sleep apnea syndrome in multiple sclerosis: report of two cases. Acta neurologica Scandinavica 88 (1993) 401-5.

[16] E.A. Marin-Sanabria, N. Kobayashi, S. Miyake, and E. Kohmura, Snoring associated with Ondine's curse in a patient with brainstem glioma. Journal of clinical neuroscience : official journal of the Neurosurgical Society of Australasia 13 (2006) 370-3.

[17] K. Ramar, Central alveolar hypoventilation and failure to wean from the ventilator. J Clin Sleep Med 5 (2009) 583-5.

[18] J.L. Pedroso, R.F. Baiense, A.P. Scalzaretto, P.B. Neto, A.F. Teixeira de Gois, and M.E. Ferraz, Ondine's curse after brainstem infarction. Neurology India 57 (2009) 206-7.

[19] H.T. Ho, P. Thajeb, and C.C. Lin, Ondine's curse in a patient with unilateral medullary and bilateral cerebellar infarctions. J Chin Med Assoc 68 (2005) 531-4.

[20] S.G. Kapnadak, I. Mikolaenko, K. Enfield, D.R. Gress, and B.R. Nathan, Ondine's curse with accompanying trigeminal and glossopharyngeal neuralgia secondary to medullary telangiectasia. Neurocritical care 12 (2010) 395-9.

[21] M.S. Nogues, K. Heidel, and E. Benarroch, Unilateral focal lesions in the rostrolateral medulla influence chemosensitivity and breathing measured during wakefulness, sleep, and exercise. Journal of neurology, neurosurgery, and psychiatry 69 (2000) 138-9.

[22] M. Habek, and V.V. Brinar, Central sleep apnea and ataxia caused by brainstem lesion due to chronic neuroleptospirosis. Neurology 73 (2009) 1923-4.

[23] M. Mendoza, and J.G. Latorre, Pearls and oy-sters: reversible Ondine's curse in a case of lateral medullary infarction. Neurology 80 (2013) e13-6.

[24] E. Fiedler, and R. Gill, Teaching NeuroImages: Ondine curse syndrome caused by dorsolateral medullary stroke. Neurology 94 (2020) e1557-e1558.

[25] P. Schestatsky, and L.N. Fernandes, Acquired Ondine's curse: case report. Arquivos de neuro-psiquiatria 62 (2004) 523-7.

[26] J. Bogousslavsky, R. Khurana, J.P. Deruaz, J.P. Hornung, F. Regli, R. Janzer, and C. Perret, Respiratory failure and unilateral caudal brainstem infarction. Annals of neurology 28 (1990) 668-73.

[27] T. Armangue, A. Macaya, E. Vazquez, M.J. Jurado, and M. Roig-Quilis, Central hypoventilation and brainstem dysgenesis. Pediatric neurology 46 (2012) 257-9.

[28] M. Mishina, S. Ohkubo, N. Kamiya, A. Abe, S. Suda, M. Sakamaki, S. Kominami, T. Mizunari, S. Kobayashi, and Y. Katayama, Efficacy of tracheostomy for central alveolar hypoventilation syndrome caused by lateral medullary infarction. Journal of Nippon Medical School = Nippon Ika Daigaku zasshi 81 (2014) 276-84.

[29] E.C. Dooling, and E.P. Richardson, Jr., Ophthalmoplegia and Ondine's curse. Arch Ophthalmol 95 (1977) 1790-3.
